# Therapeutic Potential of Synthetic Human *β*-Defensin 1 Short Motif Pep-B on Lipopolysaccharide-Stimulated Human Dental Pulp Stem Cells

**DOI:** 10.1155/2022/6141967

**Published:** 2022-01-24

**Authors:** Jue Shi, Zihe Hu, Yanyan Zhou, Minghao Zuo, Haiyan Wu, Wenjing Jin, Zhijian Xie

**Affiliations:** Stomatology Hospital, School of Stomatology, Zhejiang University School of Medicine, Clinical Research Center for Oral Diseases of Zhejiang Province, Key Laboratory of Oral Biomedical Research of Zhejiang Province, Cancer Center of Zhejiang University, Hangzhou 310006, China

## Abstract

Dental pulp inflammation is a widespread public problem usually caused by caries or trauma. Alleviating inflammation is critical to inflamed pulp repair. Human *β*-defensin 1 short motif Pep-B is a cationic peptide that has anti-inflammatory, antibacterial, and immunoregulation properties, but its repair effect on human dental pulp stem cells (hDPSCs) under inflammation remains unclear. In this study, we aimed to investigate anti-inflammatory function of Pep-B and explore its therapeutic potential in lipopolysaccharide-(LPS-) induced hDPSCs. CCK-8 assay and transwell assay evaluated effects of Pep-B on hDPSC proliferation and chemotaxis. Inflammatory response in hDPSCs was induced by LPS; after Pep-B application, lactate dehydrogenase release, intracellular ROS, inflammatory factor genes expression and possible signaling pathway were measured. Then, osteo-/odontoblast differentiation effect of Pep-B on LPS-induced hDPSCs was detected. The results showed that Pep-B promoted hDPSC proliferation and reduced LPS-induced proinflammatory marker expression, and western blot result indicated that Pep-B inhibited inflammatory activation mediated by NF-*κ*B and MAPK pathways. Pep-B also enhanced the expression of the osteo-/odontogenic genes and proteins, alkaline phosphatase activity, and nodule mineralization in LPS-stimulated hDPSCs. These findings indicate that Pep-B has anti-inflammatory activity and promote osteo-/odontoblastic differentiation in LPS-induced inflammatory environment and may have a potential role of hDPSCs for repair and regeneration.

## 1. Introduction

There are about 36% of the population has permanent tooth decay, and 9% of the population has deciduous tooth decay in the world [1]. If caries is not intervened in time, oral pathogenic microorganism will further invade the dental pulp, and bacteria lipopolysaccharide (LPS) stimulates cells to secrete proinflammatory factors causing dental inflammatory response [2, 3]. Then, human dental pulp stem cells (hDPSCs), served as reserve cells for dental pulp restoration, are recruited and migrate to the affected site and differentiate into odontoblasts to replace the damaged odontoblasts [4–6]. Odontoblasts produce beta-defensin and nitric oxide to fight against bacterial invasion and secrete extracellular matrix components to form repaired dentin and protect pulp vitality [7]. Inflammation regulation is a prerequisite for tissue healing and pulp regeneration [8]. Early reduction of inflammation level is critical to the repair and regeneration of pulp tissue [9], while excessive long-term inflammation causes cell death and tissue destruction, leading to irreversible pulp damage and serious consequences such as dental pulp necrosis, periapical disease, combined endodontic-periodontal lesions, and even tooth extraction [10]. Therefore, controlling pathogenic factors timely, alleviating the inflammatory condition to improve the self-repair capacity of hDPSCs, and promoting the restoration of pulp environment homeostasis for pulp healing have always been the focus of the treatment of oral pulp diseases.

Human *β*-defensin 1 (HBD1) is a cationic antimicrobial peptide that exists naturally in the human body [11, 12] and has important functions such as broad-spectrum antibacterial activity, inflammation inhibition, differentiation regulation, anticancer effect, and immune chemotaxis [13, 14]. HBD1 is widely present in the oral cavity (including gums, tongue, salivary gland mucosa, and other epithelial tissues) and provides a first line of defense in oral tissues [15, 16]. As a key element of the innate immune system, HBD1 exhibits high activity against Gram-negative bacteria and can control microbial community adherence to epithelial surfaces [17, 18]. Some genetic variation of the DEFB1 gene may lead to the variable expression of HBD1, which is associated with a higher susceptibility to bacterial colonization and leads to an increased risk of dental caries periodontitis and oral infections [19–21]. In addition, HBD1 may play a role in dental pulp. Studies show a high basal and continuous HBD1 expression in healthy dental pulps and significantly increase in inflamed dental pulp [22–24]. The above research results indicated that HBD1 not only maintains the ecological balance of dental pulp under physiological conditions but also plays a key role in host defense of human dental pulp against infection or microbial invasion.

Short peptides corresponding to their antimicrobial motifs are faster and easier synthesis, modification, and optimization making it an ideal candidate to accelerate their clinical transformation and applicability, and no doubt that studies about short peptides have been received extensive research interest. Based on existing research, in this study, a short HBD1 peptide that consists of 15 amino acids (ACPIFTKIQGTCYRG, Pep-B) is synthesized, which has been verified to have considerable antibacterial activity comparable to full-length proteins. However, its inhibiting inflammation effect and mechanism on LPS-stimulated hDPSCs remain unclear. Collectively, this in vitro study is aimed at investigating the anti-inflammatory effect and differentiation characteristics of exogenous Pep-B in LPS-stimulated hDPSCs and to provide experimental basis for mechanism of Pep-B in the inflamed pulp repair and application of Pep-B in endodontic disease clinically.

## 2. Materials and Methods

### 2.1. Peptide Preparation

Peptides were synthesized commercially from Sangon Biotech Co., Ltd. (Shanghai, China). The final chimeric sequences purity was examined by High Performance Liquid Chromatography (HPLC; SHIMADZU, Japan). LC-mass spectrometry (LC-MS; Thermo Fisher LTQ Orbitrap Elite) and Raman spectrum (Renishaw, United Kingdom) were used to characterize Pep-B. These lyophilized peptides were stored at −80°C and diluted in sterile ultrapure water when used.

### 2.2. HDPSC Isolation, Culture, and Characterization

All the experiments were performed with the approval of the Ethics Committee of Zhejiang Stomatology Hospital (approval number: 2018013). Briefly, freshly extracted healthy premolars for orthodontic therapy from healthy patients (18-25 years old) were collected. The dental pulp tissue was obtained using dental pincette, then cut into pieces, and digested with 2 mg/ml collagenase type I (Sigma-Aldrich, St. Louis, MO, USA) for 30 min at 37°C, cultured with DMEM containing 10% fetal bovine serum (FBS, Gibco, USA) at 37°C in 5% CO_2_. The surface markers of hDPSCs were evaluated by flow cytometry. In brief, cells were digested with 0.25% trypsin and incubated with following antibodies for 30 min: anti-CD34-PE, anti-CD45-PE, anti-CD29-PE, anti-CD90-PE, anti-CD105-PE, and anti-CD146-PE (Biolengend, USA). The stained cells were analyzed using the flow cytometry (BD bioscience Pharmingen, CytoFLEX LX, USA) to detect fluorescence intensity and positive rate.

### 2.3. Proliferation and Transwell Assays

Cell Counting Kit-8 (CCK8, Beyotime, China) was used to test cell viability. Briefly, 2 × 10^3^ cells/well were plated in 96-well plates and incubated overnight. Then, the cells were exposed to 1.25, 2.5, 5, and 10 *μ*g/ml Pep-B or control (*α*-MEM containing 10% FBS). After incubation for 1, 3, or 5 days, CCK-8 was added to each well, and the plates were further incubated for 2 h; the optical density (OD) value of each well was measured at 450 nm using a microplate reader (Perkin-Elmer, USA).

Transwell migration assays were performed to assess the chemotaxis ability of hDPSCs. The hDPSCs were suspended in serum-free medium and 200 *μ*L cell suspension containing 5 × 10^4^ cells that were plated in the upper chamber of transwell plate (8 *μ*m pore size, 24-well; Corning, CA, United States). 800 *μ*L of 10% FBS/*α*-MEM containing Pep-B (1.25, 2.5, 5, 10 *μ*g/ml Pep-B) was added in the lower chamber, and 10% FBS/*α*-MEM served as the control group. Cells were cultured for 24 and 48 h and washed with PBS for three times. Cotton swabs were used to scraped off cells on the upper side of the insert. And the cells on the underside of the membrane were fixed with methanol and stained with Crystal Violet Staining Solution (Beyotime, China). Three fields were randomly selected to count cell numbers using a light microscope. ImageJ software (National Institute of Health) was used to calculate cell number per field.

According to the results of the above experiments, the concentration of 2.5 and 5 *μ*g/ml Pep-B was selected for further experiments.

### 2.4. Lactate Dehydrogenase (LDH) Assay

Leakage of LDH in the culture medium was measured by LDH assay kit (Beyotime, China) to assess the cytotoxicity of treated hDPSCs. Briefly, hDPSCs were treated with 1 *μ*g/ml LPS (Escherichia coli 0111:B4, Sigma-Aldrich; St. Louis, MO) in the presence or absence of Pep-B (2.5, 5 *μ*g/ml) for 48 h, and the cell culture medium was collected and centrifuged to obtain the cell-free supernatants. 120 *μ*l of sample medium was mixed with 60 *μ*l reaction reagents and incubated in dark at room temperature for 30 min, and absorbance was determined at 490 nm using a microplate reader.

### 2.5. Reactive Oxygen Species (ROS) Assay

20,70-dichlorofluorescin diacetate dye (DCFH-DA) (Beyotime, China) was used to evaluated intracellular reactive oxygen species (ROS) of hDPSCs. Briefly, 2 × 10^4^ cells/well were seeded on 24 well plate and cultured overnight, then treated with 1 *μ*g/ml LPS in the presence or absence of Pep-B (2.5, 5 *μ*g/ml) for 24 h. 10 *μ*M DCFH-DA added to cells and incubated at 37°C for 30 min. After washing with PBS for three times, fluorescent microscope (Nikon, Japan) was used to take images. The fluorescent density of images was quantified using ImageJ software (National Institute of Health).

### 2.6. Quantitative Real-Time PCR (RT-qPCR)

To evaluate the capability of Pep-B in countering inflammation, hDPSCs were exposed to 1 *μ*g/ml LPS for 24 h with/without Pep-B (2.5 and 5 *μ*g/ml) to determine mRNA levels of IL6, TNF-*α*, IL8, MCP-1, COX2, and PEG2. The mRNA levels of BMP2, COL І, OPN, OSX, DSPP, and DMP1 for 7 and 14 days were performed to prove osteo-/odontogenesis ability of Pep-B under inflammation condition. TRIzol (Invitrogen, Carlsbad, CA, USA) was used to extract total RNA following the manufacturer's instructions. Then, 500 ng RNA was converted to cDNA using the transcriptor first-strand complementary DNA synthesis kit (Takara, Japan). The RT-qPCR was carried out with a SYBR Green System (Takara, Japan) according to the manufacturer's protocol. Target gene expressions were normalized with GAPDH. The primers sequences (sangon, Shanghai, China) are listed in Table 1.

### 2.7. Western Blot Analysis

HDPSCs were seeded onto six well plates (1 × 10^5^ cells per well). To analyze the potential mechanism of Pep-B inhibiting inflammation, when the cell density reaches 80%, hDPSCs were treated with LPS with or without either 2.5 or 5 *μ*g/ml Pep-B for 2 h. To evaluate the osteogenic and odontogenic differentiation of hDPSCs, cells were cultured in osteogenic induction medium with LPS with or without either 2.5 or 5 *μ*g/ml Pep-B for 14 days. Cells were lysed in RIPA buffer containing protease inhibitors (Solarbio, Beijing). A BCA Protein Assay Kit (Beyotime, China) measured protein concentration. 20 *μ*g protein of each sample was separated on 10% SDS-PAGE gels and transferred to PVDF membranes (Millipore, Bedford, MA, USA) at 350 mA V for 75 min. After blocking in 5% nonfat milk for 1 h at room temperature, the membranes were incubated with primary antibodies against anti-NF-*κ*B p65 (1 : 1000; Abcam), ERK mitogen-activated protein kinase (MAPK), phospho-ERK MAPK (1 : 1000; Cell Signaling Technology), p38 MAPK, phospho-p38 MAPK, JNK MAPK, phospho-JNK MAPK (1 : 1000; Immunoway), COL І (1 : 1000; Proteintech), and DSPP (1 : 200; Santa Cruz) overnight. *β*-Actin (1 : 5000; Proteintech) was used as a control. Next, the membranes were washed with TBST for three times, incubated with horseradish peroxidase-conjugated secondary antibody (1 : 8000; Proteintech) for 1 h at room temperature, and washed thrice with TBST. Then, the membranes were detected using enhanced chemiluminescent (NCM biotech, China). ImageJ software was used to quantify the density of protein bands.

### 2.8. Immunofluorescence Staining

HDPSCs were seeded onto 12 well glass coverslips (2 × 10^4^ cells per well) for 24 h, and then hDPSCs were treated with LPS with or without either 2.5 or 5 *μ*g/ml Pep-B for 2 h. The cells were fixed in 4% paraformaldehyde for 15 min, permeabilized with 0.05% Triton X-100 for 10 min, and blocked using 5% BSA in 37°C for 1 h. Next, cells were incubated with anti-NF-kBp65 (1 : 300; Abcam) in 4°C overnight. After washing with PBS for three times, the cells were incubated with secondary antibodies goat anti-rabbit Alexa Fluor 488 (1 : 200, Abcam) at room temperature for 1 hour, and then DAPI (5 *μ*g/ml, Solarbio) was used to stain nuclei. Images were obtained using a confocal laser scanning microscopy (Leica, Wetzlar, Germany).

### 2.9. Alkaline Phosphatase (ALP) Analysis and Alizarin Red Staining

HDPSCs (1 × 10^5^ cells/well) were seeded in six well plates and cultured with osteo-/odontogenic differentiation medium (*α*-MEM, 10% FBS, 50 *μ*g/ml ascorbic acid, 10 mmol/L *β*-glycerophosphate, 10 nmol/L dexamethasone; all reagents were purchased from Sigma-Aldrich) and treated with LPS in the presence or absence of Pep-B (2.5, 5 *μ*g/ml), and cells cultured with only differentiation medium were as the control group. The medium was replaced every 2 days.

After incubation for 7 and 14 days, ALP staining was performed using BCIP/NBT alkaline phosphatase colorimetry kit (Beyotime, China) according to the manufacturer's instructions, and photos were taken using a digital camera. ALP activity was measured by an Alkaline Phosphatase Assay Kit (LabAssayTM, Japan). BCA Protein Assay Kit (Beyotime, China) determined the total protein content to normalize ALP activity.

Mineralized nodule formation was examined by 2% alizarin red (pH 4.2) (Beyotime, China) after osteoblast differentiation for 14 days, and a digital camera was used to take photographs. To quantify alizarin staining, 10% cetylpyridinium chloride (CPC; Sigma) dissolved the mineral deposition, and OD values was measured at 562 nm.

### 2.10. Statistical Analysis

All experiments were performed for three times. The results were expressed as the mean ± standard deviation. Shapiro-Wilk test in SPSS software (SPSS Inc., Chicago, IL, United States) was used to test normality of data. The statistical significance of data was assessed by one-way analysis of variance (ANOVA), followed by the Newman–Keuls test, and the *P* < 0.05 was considered as statistical significance. The chart bars were drawn using GraphPad Prism 8.0 (GraphPad Software, La Jolla, CA, United States).

## 3. Results

### 3.1. Characters of Pep-B

Pep-B chromatogram was measured at 214 nm absorption, and the results showed that the purity of Pep-B was about 98% (Figure S1). The results of LC-mass spectrometry (LC-MS) showed that Pep-B was consisted of ACPIFTKIQGTCYRG (Figure S2), and this result was consistent with peptide sequence that we designed. Raman spectrum was performed to detect secondary structure of Pep-B, and the bands at 1240 cm^−1^, 1340 cm^−1^, 1430 cm^−1^, and 1666 cm^−1^ in the Raman spectrum were assigned to a coil structure, a *α*-helical conformation, the CH2CH3 deformation, and a *β*-strand structure (Figure S3).

### 3.2. Phenotype of hDPSCs

**Figure 1:**
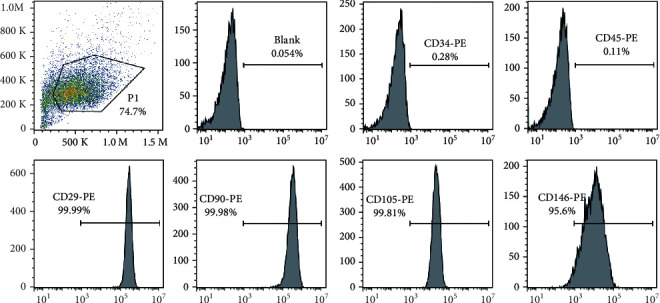
Phenotype of hDPSCs. Flow cytometric analysis showed hDPSCs strongly expressed high level of the mesenchymal stem cell (MSC) markers CD29, CD90, CD105, and CD146, but expressed negative expression of the hematopoietic cell markers CD34 and CD45.

After cultured for 7 days, cells successfully crawled out from the extracted dental pulp tissue. The flow cytometry results showed that hDPSCs expressed specific mesenchymal stem cell antigens including CD29, CD90, CD105, and CD146, while the hematopoietic cell antigens CD45 and CD34 were negative (Figure 1).

### 3.3. Pep-B Promotes the Proliferation and Chemotaxis of hDPSCs

**Figure 2:**
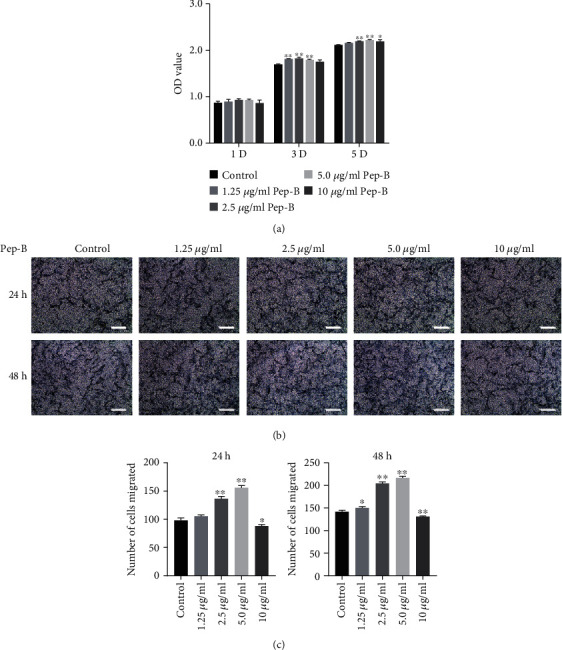
The effects of Pep-B on cell proliferation and chemotaxis of hDPSCs. Cells were treated with a series of concentrations of Pep-B (1.25, 2.5, 5, and 10 *μ*g/ml). (a) Proliferation was determined by the CCK-8 assay for 1, 3, and 5 days. (b) The migratory cells from different groups were stained by crystal violet for 24 and 48 h. (c) Statistical analysis of the average migratory cell numbers per field from different groups. ^∗^*P* < 0.05, ^∗∗^*P* < 0.01, ^∗^ represents comparison with the control group. Scale bars: 200 *μ*m.

To determine the appropriate concentration of Pep-B for in vitro study, the effect of Pep-B on hDPSC proliferation was detected using the CCK-8 assay. Cells were treated with various concentrations (1.25, 2.5, 5, or 10 *μ*g/ml) of Pep-B and *α*-MEM containing 10% FBS (control) for days 1, 3, and 5. As shown in Figure 2(a), on day 1, there was no statistical difference among Pep-B groups and control group, and on day 3, the proliferation increased markedly with 1.25, 2.5, 5 *μ*g/ml of Pep-B treatment, while 10 *μ*g/ml Pep-B unapparent decreased proliferation ability of hDPSCs. In addition, on day 5, the proliferation of hDPSCs was significantly increased by Pep-B at 2.5, 5, and 10 *μ*g/ml, and there was no statistical difference in the 1.25 *μ*g/ml Pep-B group compared to the control group. Above all, it could be concluded that Pep-B increased the proliferation ability of hDPSCs.

To investigate the effect of Pep-B on migratory capacities of hDPSCs, transwell assay was performed at 24 and 48 h. The result showed that the Pep-B group had significantly more numerous migratory cells than those in the control group apart from 10 *μ*g/ml of the Pep-B group (Figures 2(b) and 2(c)). There was no doubt that appropriate concentration of Pep-B increased the chemotaxis ability of hDPSCs.

After comprehensive analysis of the results and for convenience of the study, 2.5 and 5 *μ*g/ml of Pep-B were selected in all subsequent experiments.

### 3.4. Pep-B Ameliorates LPS-Induced Cytotoxicity and Oxidative Stress in hDPSCs

**Figure 3:**
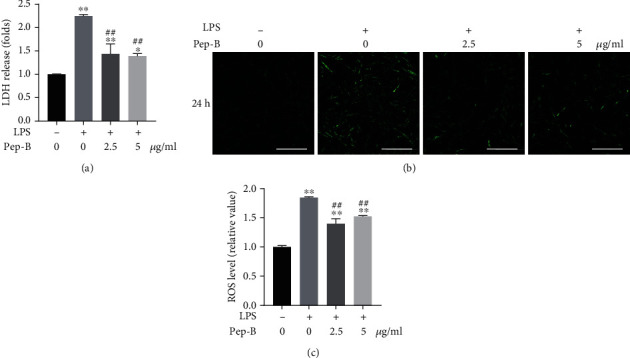
Pep-B ameliorated LPS-induced LDH release and oxidative stress in hDPSCs. Cells were treated with LPS (1 *μ*g/ml) in the presence or absence of Pep-B (2.5, 5 *μ*g/ml) for 24 h. (a) The LDH released was determined using a commercial kit. (b) Intracellular ROS was determined by DCFH-DA staining. (c) The stained images were quantified by fluorescence density from different group. ^∗^*P* < 0.05, ^∗∗^, ##*P* < 0.01, ^∗^ represents comparison with the control group, # represents comparison with the LPS group. Scale bars: 100 *μ*m.

To evaluate the effect of Pep-B on LPS-induced hDPSCs, a LDH assay was used to assess leakage of LDH from hDPSCs which were LPS-induced or treated with Pep-B. The result demonstrated LPS stimulation caused hDPSCs to release more LDH into the medium compared with that in the control group; however, Pep-B treatment reduced LDH release indicating that Pep-B could protect dental pulp cells from LPS-induced cytotoxicity (Figure 3(a)).

To examine patterns of oxidative stress, the levels of intracellular ROS in hDPSCs were detected using DCFH-DA staining. The result indicated LPS exposure remarkably increased ROS production compared with the control group, while Pep-B prevented ROS production induced by LPS (Figures 3(b) and 3(c)). The finding revealed that Pep-B played a vital role in mitigating LPS-induced oxidative stress in hDPSCs.

### 3.5. Pep-B Suppresses LPS-Induced Inflammation in hDPSCs

**Figure 4:**
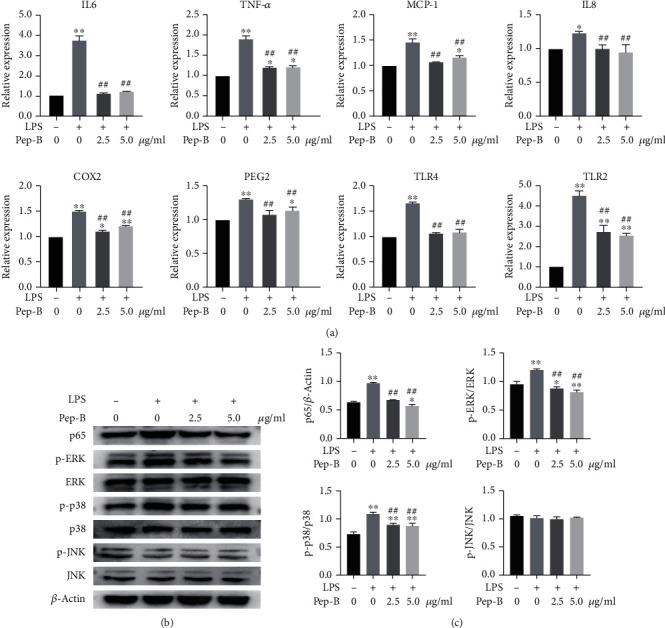
Anti-inflammatory effects of Pep-B in hDPSCs. Cells were treated with LPS (1 *μ*g/ml) in the presence or absence of Pep-B (2.5, 5 *μ*g/ml). (a) qRT-PCR analysis to quantify inflammatory marker gene expression of different groups in hDPSCs. (b) The protein expression of p65, phospho-ERK, phospho-p38, and phospho-JNK was determined by means of western blot analysis. (c) Quantification of the band density observed in (b) determined using ImageJ software. ^∗^*P* < 0.05, ^∗∗^, ##*P* < 0.01, ^∗^ represents comparison with the control group, # represents comparison with the LPS group.

To assess the ability of Pep-B to reduce the inflammation response induced by LPS, hDPSCs were treated with 1 *μ*g/ml LPS in the presence or absence of Pep-B, and cells were collected after incubation for 24 h; then, IL-6, TNF-*α*, MCP-1, IL-8, COX2, PEG2, toll-like receptor (TLR) 2, and TLR4 mRNA expression were determined by RT-qPCR. As shown in Figure 4(a), Pep-B significantly inhibited the LPS-induced inflammatory response in hDPSCs. To explore the potential underlying mechanism of Pep-B inhibiting inflammation, LPS-induced inflammation-related signaling pathway was examined. Western blot results displayed that Pep-B treatment decreased the elevated level of NF-*κ*Bp65, phosphorylated ERK, and p38 MAPK in LPS-induced hDPSCs with no significant effect on the levels of JNK (Figures 4(b) and 4(c)). Meanwhile, immunofluorescence images (Figure S4) showed that NF-*κ*Bp65 was present in the cytoplasm of untreated hDPSCs, and LPS treatment markedly increased the activation of nuclear NF-*κ*Bp65 and promoted its translocation; however, Pep-B treatment reduced translocation of nuclear NF-*κ*Bp65 compared to the LPS group. These suggested that the NF-*κ*B and MAPK signaling pathway was inhibited by Pep-B in LPS-induced hDPSCs.

### 3.6. Effects of Pep-B on the Expression of Osteo-/Odontogenic Genes and Proteins in LPS-Induced hDPSCs

To assess the effects of Pep-B on the osteo-/odontoblast differentiation of LPS-stimulated hDPSCs, cells cultured in osteogenic medium were treated or not with 2.5 and 5 *μ*g/ml Pep-B in the presence of 1 *μ*g/ml LPS for 7 and 14 days for RT-qPCR analysis and alkaline phosphatase (ALP) analysis, and 14 days for western Blot analysis and alizarin red staining (ARS), and the control group were cultured with only osteogenic medium.

**Figure 5:**
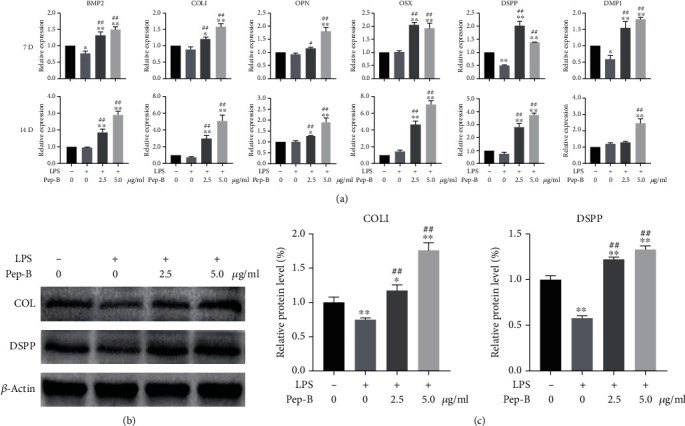
Effects of Pep-B on the expression of osteo-/odontoblast genes in LPS-induced hDPSCs. (a) The mRNA expression of BMP2, COL І, OPN, OSX, DSPP, and DMP1 was detected by qRT-PCR at 7 and 14 days. (b) The protein expression of COL І and DSPP was examined by western blot at 14 days. (c) The quantification of the western blot was determined by densitometry using ImageJ software. ^∗^, #*P* < 0.05, ^∗∗^, ##*P* < 0.01, ^∗^ represents comparison with the control group, # represents comparison with the LPS group.

When hDPSCs were induced by LPS to inflammation, the mRNA levels of osteogenic genes (BMP2, COL І, OPN, OSX) were inhibited. The odontogenic gene (DSPP and DMP1) mRNA levels were decreased under LPS-stimulated condition on days 7 and 14 as compared to those in the control group, while at 14 days, the mRNA level of DMP1 was increased with no statistic difference (Figure 5(a)). Pep-B treatment significantly improved the mRNA levels of osteogenic and odontogenic genes compared with the LPS group on days 7 and 14.

These findings were highly consistent with the western blot results. The protein levels of COL І and DSPP of hDPSCs were decreased under LPS-stimulated condition on day 14; Pep-B usage ameliorated the phenomenon and increased COL І and DSPP protein levels compared with LPS groups (Figures 5(b) and 5(c)).

### 3.7. Effects of Pep-B on the Osteogenic Differentiation and Mineralization in LPS-Induced hDPSCs

**Figure 6:**
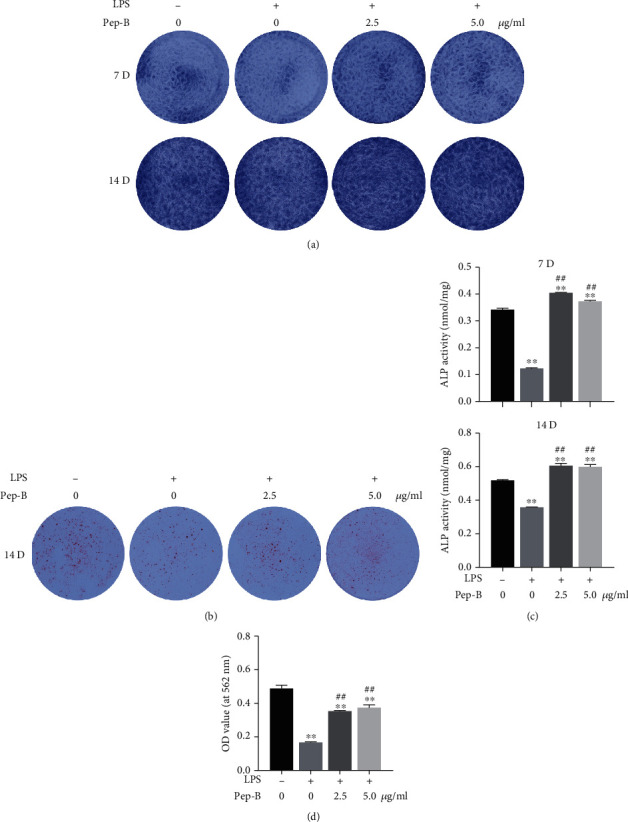
Effects of Pep-B on the osteoblast differentiation in hDPSCs. Cells were treated with LPS (1 *μ*g/ml) in the presence or absence of Pep-B (2.5, 5 *μ*g/ml) in osteo-/odontoblast induced medium. (a) ALP staining and (c) ALP activity at 7 and 14 days. (b) ARS staining and (d) mineralization nodules quantification at 14 days. ^∗∗^, ##*P* < 0.01, ^∗^ represents comparison with the control group, # represents comparison with the LPS group.

On days 7 and 14, ALP staining and activity were suppressed by LPS application, and Pep-B significantly treatment ameliorated this phenomenon as compared with that in the LPS group (Figures 6(a) and 6(c)). Consistent with that in ALP experiment, alizarin red staining showed that the mineralized areas were significantly decreased in the LPS group as compared with the control group on day 14, and the staining intensity increased after Pep-B application (Figures 6(b) and 6(d)). These findings indicated that Pep-B could improve the osteogenic ability of hDPSCs in the inflammatory condition.

## 4. Discussion

Bacterial penetration through dentinal tubule and root canal to pulp is an important risk factor for pulp inflammation, necrosis, and periapical lesions [25]. Dental pulp cells are the reserve cells for pulp injury and repair, and a process of proliferation, differentiation, and mineralization of hDPSC initiate dentin formation is essential for pulp repair and the maintenance of tooth integrity [4], while repeated stimulation of bacterial toxins (such as LPS) induce DNA double-strand breaks and DNA damage responses in hDPSCs, then have significant effect on cell proliferation and apoptosis, and attenuate the osteo-/odontoblastic differentiation potential and mineralization ability of hDPSCs [26, 27], thus greatly interfering with the formation of restorative dentin and limiting the repair of injured pulp tissues [28, 29]. Thus, removing pathogenic bacteria and reducing inflammation response timely can enable hDPSCs to preserve its stem cell properties and is essential to protect pulp tissue and promote recovery of damaged tissue. Pep-B is a cationic antimicrobial peptide with antibacterial, anti-inflammation, and immunomodulatory effects, which may be a potential agent to relieve pulp inflammation and restore pulp function. Therefore, in this study, we explored the anti-inflammation and differentiation effects of Pep-B in LPS-stimulated hDPSCs to assess its potential as a therapeutic agent for pulpitis.

LPS, as the main component of the outer membrane of Gram-negative bacteria, plays a crucial role as a virulence factor leading to inflammatory response [28, 30], attenuates the odontoblastic differentiation of hDPSCs [31, 32], and is often used in the establishment of the inflammatory microenvironment model in vitro [33, 34]. In this study, 1 *μ*g/ml LPS was used to stimulate hDPSCs to mimic an in vitro inflammatory microenvironment and to explore the anti-inflammation and differentiation function of Pep-B in vitro. When hDPSCs were stimulated with LPS, many inflammatory cytokine (including IL6, TNF-*α*, MCP-1, IL8, COX2, and PEG2) expressions increased compared with the control group, as described in previous studies [28, 32]. Our data showed that Pep-B treatment resulted a series of protective effects in LPS-induced hDPSCs. Firstly, we observed that Pep-B treatment reduced LPS-induced cytotoxicity and oxidative stress as evidenced by decreasing LPS-induced LDH release and ROS production. Secondly, we observed that Pep-B downregulated the mRNA expression of proinflammatory genes induced by LPS. Based on recent studies, the HBD family has high affinity to LPS in vitro and can directly bond to LPS, thus inhibiting inflammatory response [2, 35, 36], and these findings further support our results. Pep-B is a motif of cationic polypeptide that commonly acts on microorganisms by electrostatic binding of its positive charge with negatively charged surface constituents of bacteria, such as anionic phospholipids and phosphate groups on LPS of gram-negative bacteria [17]. Hence, we speculated that its anti-inflammatory effect was that Pep-B neutralized LPS and then reduced LPS-induced inflammatory response.

Promoting the differentiation of inflamed hDPSCs into odontoblast and forming restorative dentin is the key of regulating pulp inflammation and promoting pulp repair [37]. Therefore, we further explored the effect of Pep-B on restorative ability of dental pulp cells under inflammatory condition. Our study found that PEP-B ameliorated the decreased expression of osteogenic/odontogenic genes and proteins in LPS-induced hDPSCs, enhanced ALP activity, and increased mineralized nodule formation. These results suggested that Pep-B could promote the differentiation of hDPSCs in an inflammatory environment. And this is consistent with existing studies that the HBD family can promote the differentiation of some stem cells and precursor cells in normal or inflammatory environment [38]. The combination of HBD3 and gold nanoparticles can promote the differentiation of human periodontal ligament stem cells into osteoblasts under inflammation [39]. HBD4 inhibited the LPS-induced inflammatory response and induced the differentiation of SHED into odontoblasts and osteoblasts [2]. In conclusion, Pep-B not only can reduce the inflammatory response but also facilitates cell differentiation and remineralizing process in inflamed hDPSCs, which may be a potential therapeutic agent for the dental pulp tissue regeneration under inflammatory conditions.

Pep-B shows a clear advantage in inhibiting inflammation in LPS-induced hDPSCs, but the mechanism is not fully understood. Next, we attempted to determine related signaling pathway of Pep-B. Inflammatory effects induced by LPS are mediated by several different intracellular signaling pathways, including NF-*κ*B and MAPK signaling pathway [40]. When cells were exposed to stimuli (like LPS and inflammatory cytokines), NF-*κ*B and MAPK signaling pathway are activated and stimulate the expression of proinflammatory cytokines [41, 42]. However, suppression of NF-*κ*B and MAPK signaling pathway can have an important role in reducing inflammation. Study showed that the inhibition of NF-*κ*B and MAPK signaling could suppress inflammatory mediators and cytokine production and enhanced odontoblastic differentiation and collagen matrix formation, which may be a potential intervention to promote pulp tissue regeneration [29, 43]. Our findings are consistent with the above studies. In our study, western blot results showed that Pep-B treatment inhibited the activation of NF-*κ*B and reduced the phosphorylation level of ERK and p38 MAPK in LPS-stimulated hDPSCs. This suggested that the anti-inflammation effect of Pep-B on LPS-induced hDPSCs is related to NF-*κ*B pathway inhibition and reduction of ERK and p38 MAPK signaling pathway activation.

However, there are still some questions that need to be explored. In future experiments, we will have an indepth study: In future experiments, (1) we will examine the anti-inflammation effect of Pep-B in other cell lines in dental pulp (such as fibroblasts and lymphocytes) to comprehensively evaluate the role of Pep-B in controlling inflammation and hard tissue regeneration; (2) we will further establish in vivo model to clarify the anti-inflammation and osteo-/odontoblastic differentiation effect of Pep-B, determine the optimal concentration for in vivo application, and improve its stability through modifying structure or using suitable scaffold materials.

## 5. Conclusion

In this study, we reveal that Pep-B treatment can not only reduce inflammation but also promote osteo-/odontoblastic differentiation in LPS-stimulated hDPSCs. Our findings suggest new therapeutic strategies that Pep-B may hold great potential in inflammation control and preservation of dental pulp tissue.

## Figures and Tables

**Table 1:** qPCR genes and primer sequences.

Gene (human)	Primer	Sequence (5′-3′)
IL6	Forward	CAGGGAGAGGGAGCGATAAAC
	Reverse	CAGGGAGAAGGCAACTGGAC
TNF-*α*	Forward	ACATGCCTACACCTACCTGC
	Reverse	TGAACATCTCTGCTCGTCGC
MCP-1	Forward	TCAAACTGAAGCTCGCACTCTCG
	Reverse	GGGAATGAAGGTGGCTGCTATGAG
IL8	Forward	AACTGAGAGTGATTGAGAGTGG
	Reverse	ATGAATTCTCAGCCCTCTTCAA
COX2	Forward	TGTCAAAACCGAGGTGTATGTA
	Reverse	AACGTTCCAAAATCCCTTGAAG
PEG2	Forward	TTCTGAGACTAATGCGTTCAGT
	Reverse	TTACTGGCATCTGACTGTGTAG
TLR2	Forward	AAGCAGCATATTTTACTGCTGG
	Reverse	CCTGAAACAAACTTTCATCGGT
TLR4	Forward	GACTGGGTAAGGAATGAGCTAG
	Reverse	ACCTTTCGGCTTTTATGGAAAC
BMP2	Forward	AGAATGCAAGCAGGTGGGAA
	Reverse	TTCCGCTGTTTGTGTTTGGC
COL І	Forward	TCTAGACATGTTCAGCTTTGTGGAC
	Reverse	TCTGTACGCAGGTGATTGGTG
OPN	Forward	TCTGGGAGGGCTTGGTTGTC
	Reverse	TTTCCTTGGTCGGCGTTTG
OSX	Forward	AGTCAGAGTAGGACTGTAGGAC
	Reverse	GCCATAGTGAACTTCCTCCTCA
DSPP	Forward	GCTGGAAGCAATAACAGTACAG
	Reverse	TGCTGTTGATCTGAGGTGTTAT
DMP1	Forward	CAAAGAAGATAGCAACTCCACG
	Reverse	CATCAACTGTTAATTTCCGGCT
GAPDH	Forward	GCTCTCTGCTCCTCCTGTTC
	Reverse	GACTCCGACCTTCACCTTCC

## Data Availability

The original data used to support the findings of this study are included within the article.

## Supplementary Materials

Supplementary Materials Figure S1: example of a synthesized Pep-B chromatogram measured at 214 nm absorption. The purity was calculated using peak area ratio: 2060318/2083537 × 100% ≈ 98.86%. Figure S2: the fragmentation spectrum of a synthesized Pep-B using LC-mass spectrometry (LC-MS). The results showed that Pep-B was consisted of ACPIFTKIQGTCYRG. Figure S3: Raman spectrum to detect secondary structure of Pep-B. The bands at 1240 cm^−1^, 1340 cm^−1^, 1430 cm^−1^, and 1666 cm^−1^ in the Raman spectrum were assigned to a coil structure an *α*-helical conformation, the CH2CH3 deformation, and a *β*-strand structure. Figure S4: effect of Pep-B on NF-*κ*Bp65 translocation in LPS-treated hDPSCs. Cells were treated with LPS (1 *μ*g/ml) in the presence or absence of Pep-B (2.5, 5 *μ*g/ml) for 2 h and then subjected to immunofluorescence analysis. Scale bars: 50 *μ*m.
