# Supplementary material for: Therapeutic Potential of Synthetic Human β-Defensin 1 Short Motif Pep-B on Lipopolysaccharide-Stimulated Human Dental Pulp Stem Cells
Source: Mediators Inflamm. 2022 Jan 24;2022:6141967. doi: 10.1155/2022/6141967 (PMC8803462; doi:10.1155/2022/6141967)
Supplement: Supplementary Materials — Figure S1: example of a synthesized Pep-B chromatogram measured at 214 nm absorption. The purity was calculated using peak area ratio: 2060318/2083537 × 100% ≈ 98.86%. Figure S2: the fragmentation spectrum of a synthesized Pep-B using LC-mass spectrometry (LC-MS). The results showed that Pep-B was consisted of ACPIFTKIQGTCYRG. Figure S3: Raman spectrum to detect secondary structure of Pep-B. The bands at 1240 cm−1, 1340 cm−1, 1430 cm−1, and 1666 cm−1 in the Raman spectrum were assigned to a coil structure an α-helical conformation, the CH2CH3 deformation, and a β-strand structure. Figure S4: effect of Pep-B on NF-κBp65 translocation in LPS-treated hDPSCs. Cells were treated with LPS (1 μg/ml) in the presence or absence of Pep-B (2.5, 5 μg/ml) for 2 h and then subjected to immunofluorescence analysis. Scale bars: 50 μm. [file 6141967.f1.docx]

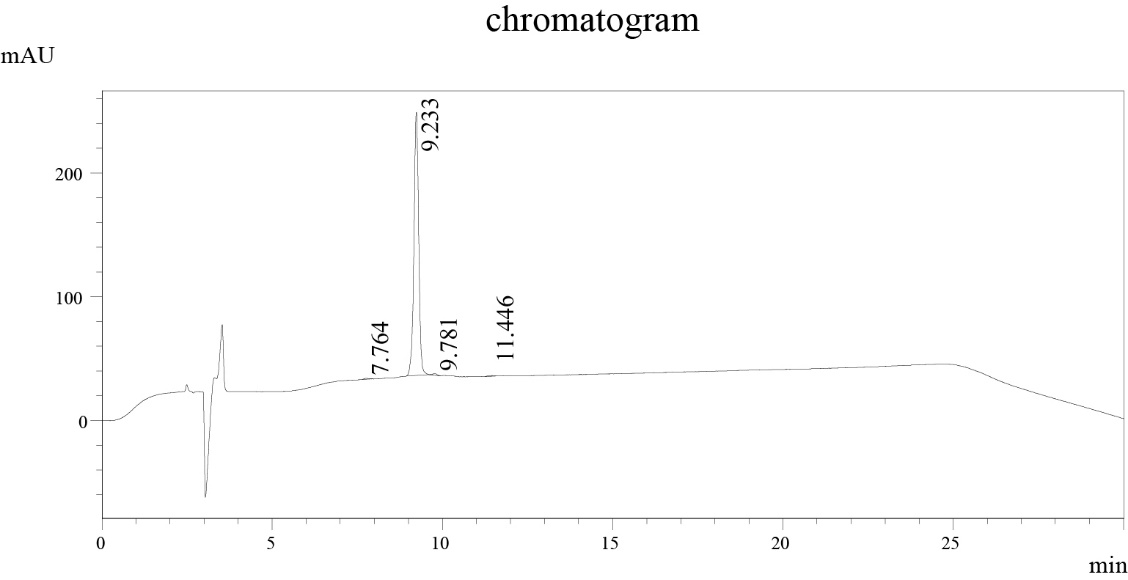


**Peak Table**

| **Peak** | **Ret.Time** | **Area** | **Height** |
| --- | --- | --- | --- |
| 1 | 7.764 | 5715 | 733 |
| 2 | 9.233 | 2060318 | 212438 |
| 3 | 9.781 | 11360 | 1197 |
| 4 | 11.446 | 6144 | 577 |
| Total |  | 2083537 | 214944 |

**Figure S1.** Example of a synthesized Pep-B chromatogram measured at 214 nm absorption. The purity was calculated using peak area ratio: 2060318 / 2083537 ⅹ 100% ≈ 98.86%.


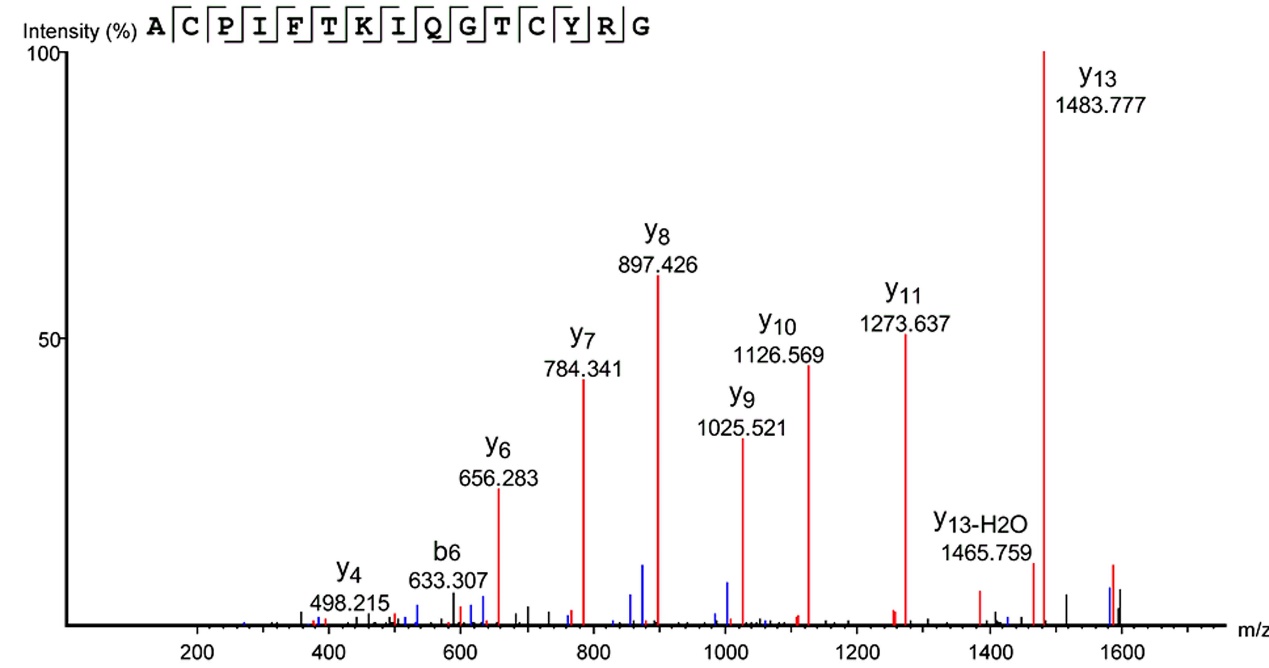


**Figure S2.** The fragmentation spectrum of a synthesized Pep-B using LC-mass spectrometry (LC-MS). The results showed that Pep-B was consisted of ACPIFTKIQGTCYRG.


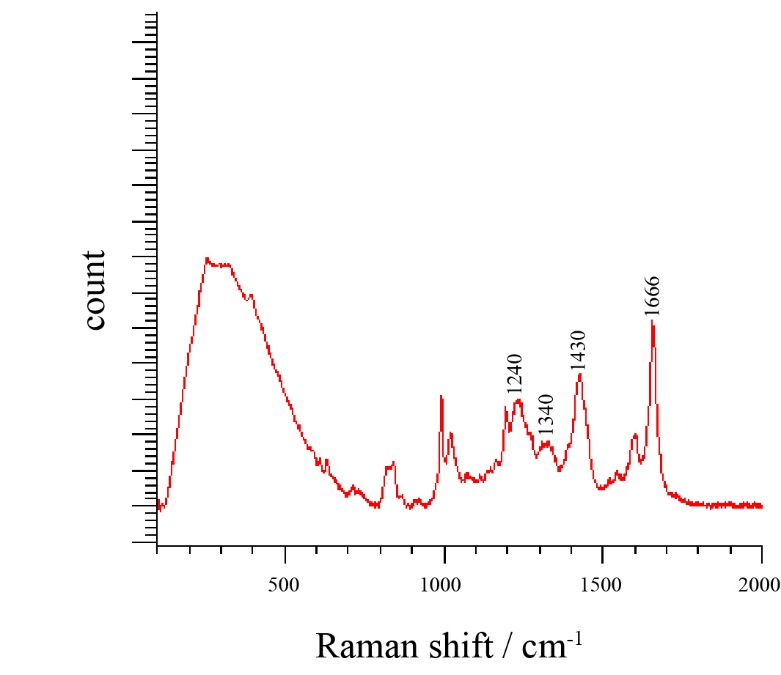


**Figure S3.** Raman spectrum to detect secondary structure of Pep-B. The bands at 1240 cm^-1^, 1340 cm^-1^, 1430 cm^-1^ and 1666 cm^-1^ in the Raman spectrum were assigned to a coil structure, an α-helical conformation, the CH2CH3 deformation and a β-strand structure.


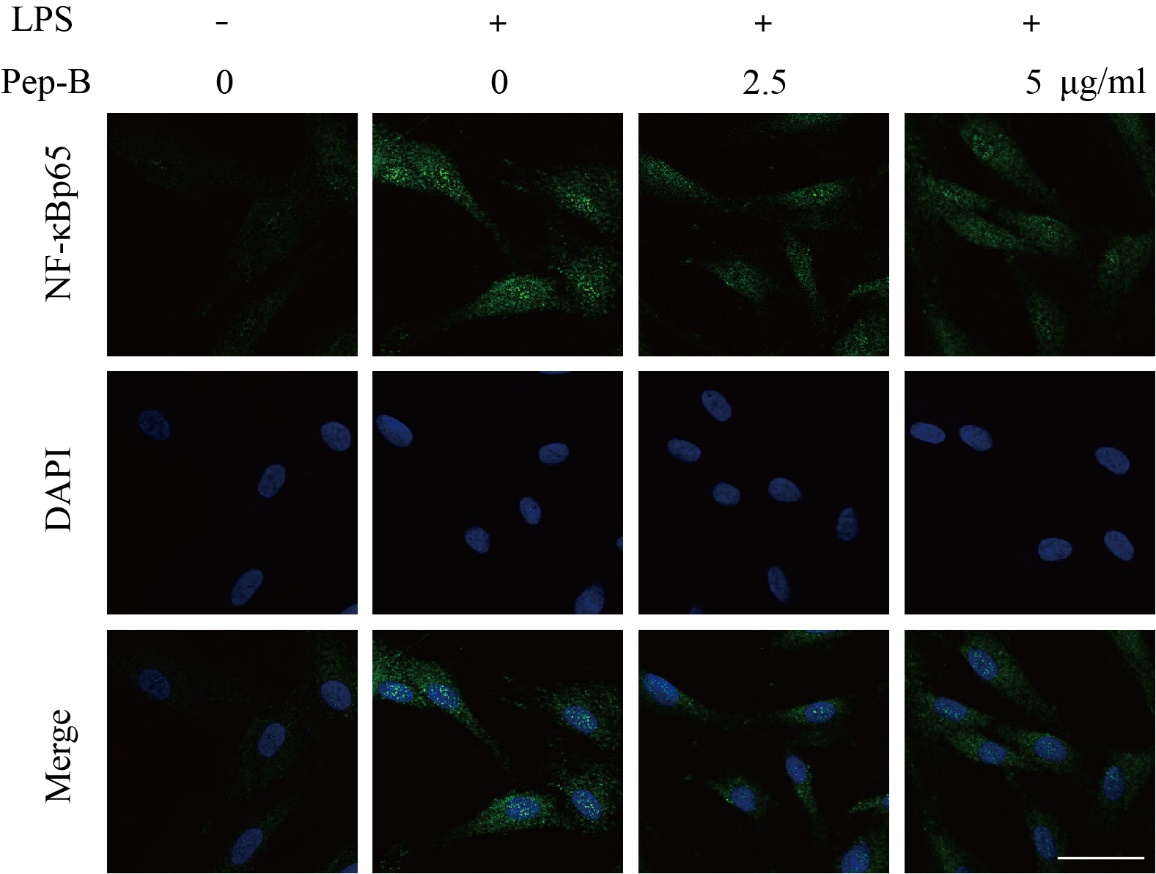


**Figure S4.** Effect of Pep-B on NF-κBp65 translocation in LPS-treated hDPSCs. Cells were treated with LPS (1 μg/ml) in the presence or absence of Pep-B (2.5, 5 μg/ml) for 2 h and then subjected to immunofluorescence analysis. Scale bars: 50 μm.
